# Offer of a Bribe for a Good Review by a Dentist

**Published:** 1847-06

**Authors:** Edward Miles


*Offer of a Bribe for a good Review by a Dentist.*—15, Liverpool-street, City. Edward Miles presents his respects to the Editor of the *Medical Times,* if he sends his work to him, which he has recently published, will it be reviewed in the *next or following number?* E. M's reason for inquiry is this: he has been dissappointed in some cases; the review has been put off, or else some trifling notice *only* has been taken of it, from which *no extract could be taken.* All E. M. asks for is an immediate, a fair, conscientious, and honest report of it. The title is "Health, Comfort, and Longevity promoted, &c." Sixty pages. £10 will be enclosed.

The principle on which E. M. encloses a trifling sum is because he does not consider it reasonable to suppose that an editor can give up his valuable time without some small remuneration. At the same time E. M. acknowledges it would be more agreeable to his feelings, if it would answer as well, if he engaged for six insertions of an advertisement following the review. In that case the ten shillings which would be sent might be placed to E. M's credit.

A word, per return of post, will very much oblige, just to say whether, if E. M. sends the book, it will be reviewed *per very next number of the Medical Times.*

My plan is to take an extract from the review, if it is so favorable to allow me, and to *then* advertise the work.

Thy friend, very respectfully,

EDWARD MILES.

9th month, 16, 1846.

